# Diffuse colonies of human skin fibroblasts in relation to cellular senescence and proliferation

**DOI:** 10.18632/aging.101240

**Published:** 2017-05-16

**Authors:** Vadim Zorin, Alla Zorina, Nadezhda Smetanina, Pavel Kopnin, Ivan V. Ozerov, Sergey Leonov, Artur Isaev, Dmitry Klokov, Andreyan N. Osipov

**Affiliations:** 1 Human Stem Cells Institute, Moscow 119333, Russia; 2 State Research Center - Burnasyan Federal Medical Biophysical Center of Federal Medical Biological Agency, Moscow 123098, Russia; 3 Blokhin Cancer Research Center, Moscow 115478, Russia; 4 Moscow Institute of Physics and Technology, Dolgoprudny, Moscow Region 141700, Russia; 5 Institute of Cell Biophysics, Russian Academy of Sciences, Pushchino, Moscow Region 142290, Russia; 6 Canadian Nuclear Laboratories, Chalk River, Ontario K0J1J0, Canada; 7 Semenov Institute of Chemical Physics, Russian Academy of Sciences, Moscow 119991, Russia

**Keywords:** skin fibroblasts, clonogenic assay, proliferation, cellular senescence, β-galactosidase

## Abstract

Development of personalized skin treatment in medicine and skin care may benefit from simple and accurate evaluation of the fraction of senescent skin fibroblasts that lost their proliferative capacity. We examined whether enriched analysis of colonies formed by primary human skin fibroblasts, a simple and widely available cellular assay, could reveal correlations with the fraction of senescent cells in heterogenic cell population. We measured fractions of senescence associated β-galactosidase (SA-βgal) positive cells in either mass cultures or colonies of various morphological types (dense, mixed and diffuse) formed by skin fibroblasts from 10 human donors. Although the donors were chosen to be within the same age group (33-54 years), the colony forming efficiency of their fibroblasts (ECO-f) and the percentage of dense, mixed and diffuse colonies varied greatly among the donors. We showed, for the first time, that the SA-βgal positive fraction was the largest in diffuse colonies, confirming that they originated from cells with the least proliferative capacity. The percentage of diffuse colonies was also found to correlate with the SA-βgal positive cells in mass culture. Using Ki67 as a cell proliferation marker, we further demonstrated a strong inverse correlation (r=−0.85, p=0.02) between the percentage of diffuse colonies and the fraction of Ki67+ cells. Moreover, a significant inverse correlation (r=−0.94, p=0.0001) between the percentage of diffuse colonies and ECO-f was found. Our data indicate that quantification of a fraction of diffuse colonies may provide a simple and useful method to evaluate the extent of cellular senescence in human skin fibroblasts.

## INTRODUCTION

Fibroblasts form one the most important cellular components of the skin derma. During aging, skin fibroblasts undergo substantial changes in their functional activity, morphology and proliferative potential [1–4]. In the skin, fibroblasts are responsible for the whole range of various sophisticated functions. These include both homeostasis of the dermal intercellular matrix and maintenance of the physiological condition of other skin layers. The former is carried out by remodeling and renewing the intercellular matrix through degradation of used up dermal components and synthesis of the new ones, including collagen and elastin. The latter represents the main chain in skin metabolism [1]. The number of dermal fibroblasts decreases with aging, along with their ability to synthesize active soluble factors and to maintain proteostasis of components of the intercellular dermal matrix [2, 4]. The skin thinning, the loss of skin flexibility and elasticity, and wrinkle formation are natural consequences of such a decline. Therefore, we suggested that evaluating the proliferative potential of dermal fibroblasts is of great significance to help physicians-cosmetologists to create an optimal skin care program, as well as to predict the extent of clinical effect after application of an intradermal procedure to a patient.

Measuring the ability to form colonies *in vitro* represents one of the “gold standard” methods for the assessment of the clonogenic survival of cells [5]. The method was initially developed to evaluate the loss of reproductive capacity (reproductive death) of cells after exposure to damaging agents, particularly ionizing radiation [5]. Later it was shown that cells isolated from biopsy material from different patients had varying ability for colony formation [6]. This allows for comparative assessment of different patient's cell capacity to proliferate and may represent a promising avenue for personalized medicine. Beside a colony-forming efficiency of fibroblasts (ECO-f), defined as percentage of plated cells that are able to form colonies [7], the evaluation of colony size/type distribution [8, 9] provides additional important information especially for heterogenic cell populations such as primary fibroblasts, including mitotically active (MF) and differentiated (mature) postmitotic (PMF) fibroblasts. In this case, the size of the colony depends directly on the proliferative capacity of cell-precursors. For example, MF can be divided into the following three types: MF I, MF II, and MF III. These are defined by cells morphology, proliferative potential, and the ability to synthesize specific cytokines/growth factors [10], where the MF I cell type possesses the highest proliferative potential, undergoing about 25 – 30 cell divisions before they differentiate into the MF II cell population. Subsequently, the MF II type cells undergo about 15 – 20 cell divisions before they differentiate into MF III type cells, whereas the MF III cells undergo only 5–8 cell divisions before differentiation into PMF. Due to these differences, MF cells can form morphologically distinct colonies that can be broken down into the following three types: dense (or compact), diffuse and mixed colonies [8, 9]. If the fractions of each of these colony phenotypes are known, one can evaluate the proliferative potential of the entire fibroblasts culture using the following formula: PP = [1(DC) + 2(MC) + 3(CC)] / 100%, where PP is proliferative potential, DC, MC and CC are percentages of diffuse, mixed and compact colonies, respectively [9].

On the other hand, cellular aging, traditionally assessed by the fraction of senescence associated β-galactosidase (SA-βgal) positive cells, along with the degree of differentiation are closely associated with the proliferative capacity of cells [11]. With aging, intracellular β-galactosidase accumulates in lysosomes and a sharp increase in the β-galactosidase activity in older cells is traditionally considered to be a classic marker of cellular aging [12]. Therefore, it could be anticipated that the fraction of aging cells in colonies of the diffuse phenotype would be larger than that in the colonies of the dense phenotype. Although previous attempts to correlate colony formation ability and the size of colonies with cellular aging failed [13]. To our knowledge, there are no studies that previously examined such assumption and assessed the fraction of aging cells in colonies of various types.

Therefore, the aim of this work was to verify the assumptions regarding the relationship of cellular aging with the formation of fibroblast colonies of different phenotypes, and to examine whether such enriched analysis of colony formation may be used for evaluating the degree of cellular senescence [12]. To this end, we measured the fraction of SA-βgal positive cells (SA-βgal+) in the three types of colonies (dense, mixed and diffuse) of human skin fibroblasts from donors of various ages. We further examined correlations between the colony phenotypes and the fraction of proliferating cells that was measured using Ki67 as a marker for cellular proliferation. Ki67 protein is present in actively proliferating cells (during G1, S, G2 and M phases of the cell cycle), while being absent in resting (G0 phase) cells [14, 15]. The expression of Ki67 was shown to be associated with aging in that aging cells that lost their proliferative and colony forming capacity become Ki67-negative [16].

## RESULTS

### Clonogenic analysis

**Figure 1:**
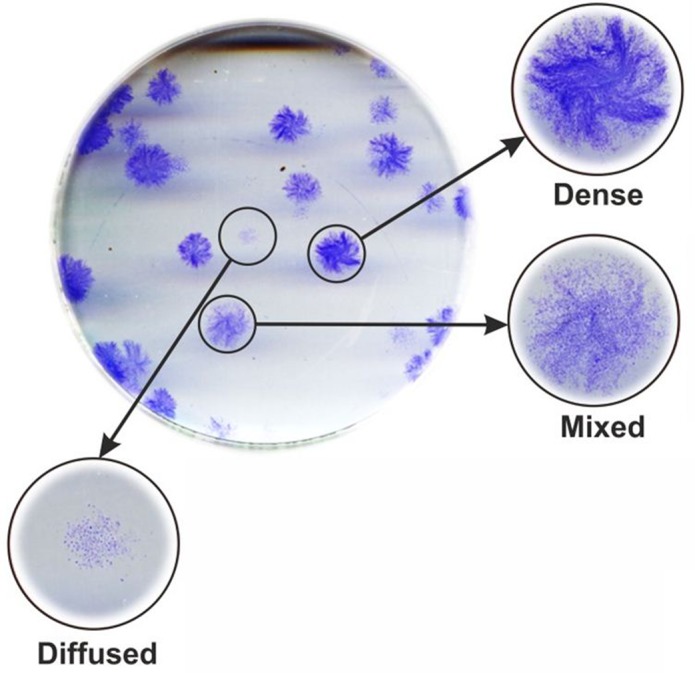
Different colony phenotypes Colonies were phenotyped based on the density of the stain. Typical examples of the three phenotypes are shown.

The primary cultures of human fibroblasts were isolated from skin biopsies taken from 10 female patients of 33 to 54 years of age, according to the standard procedures [17], and the fibroblast colonies from these cultures were phenotyped in terms of the dense, mixed and diffuse plaques (Figure 1). Although the age span of the patients was not great and is sometimes used as a single age category in aging studies [18, 19], a remarkable inter-individual variability in the clonogenic capacity of their skin fibroblasts was found as evident from the results of their clonogenic analysis (Table 1). Indeed, the variability was demonstrated both in ECO-f (from 9.7 up to 61.0%) and in the fractions of each colony phenotype. A greater range (from 8.6 up to 89.6%) was seen for the fraction of diffuse colonies that are formed by descendants of cells with a low proliferative potential. Regression analysis revealed a strong inverse correlation between ECO-f and the fraction of diffuse colonies (Table 2), indicating that colony phenotype is related to the colony formation capacity. Consequently, diffuse colonies were predominantly formed in cell cultures that have low colony formation capacity. No correlation between any of the four clonogenic parameters and the age was seen (Table 2). It follows that the clonogenic parameters studied may reflect the proliferative capacity of a fibroblast population of a particular individual and are not related to the patient's chronological age.

**Table 1:** Clonogenic analysis of human skin fibroblasts from 10 human donors

Patient ID	Patient age	ECO-f ^1^, %	Dense colonies, %	Diffuse colonies, %	Mixed colonies, %
1	38	59.0±9.0	36.3±2.9	12.7±1.9	51.0±1.0
2	33	56.7±3.4	11.7±0.5	20.2±3.6	68.1±3.1
3	33	24.7±2.7	5.5±0.6	56.5±2.0	38.0±1.4
4	54	11.0±0.3	15.1±2.6	57.7±4.8	27.2±2.2
5	48	61.0±3.0	31.9±3.8	8.6±2.9	59.5±0.9
6	34	9.7±1.0	3.1±3.0	89.6±2.1	7.3±1.0
7	43	40.3±2.4	10.9±3.1	36.9±5.3	52.2±2.2
8	46	15.0±1.7	6.5±1.5	58.0±2.0	35.5±0.5
9	43	40.3±0.4	40.1±3.9	21.4±2.8	38.5±1.2
10	41	42.0±4.0	5.4±1.9	31.5±5.4	63.2±3.6

1 ECO-f, colony-forming efficiency of fibroblasts. Values are means ± SEM calculated from three determinations (three replicate petri dishes)

**Table 2:** Correlation matrix for clonogenic parameters of human skin fibroblasts and donor age

	Patient age	ECO-f^1^, %	Fraction of dense colonies, %	Fraction of diffuse colonies, %	Fraction of mixed colonies, %
**Patient age**	1.00				
**ECO-f, %**	−0.16 p=0.66	1.00			
**Fraction of dense colonies**	0.27 p=0.45	0.59 p=0.07	1.00		
**Fraction of diffuse colonies**	−0.12 p=0.76	−0.94 p<0.01	−0.71 p=0.02	1.00	
**Fraction of mixed colonies**	−0.05 p=0.89	0.85 p<0.01	0.23 p=0.52	−0.85 p<0.01	1.00

1 ECO-f, colony-forming efficiency of fibroblasts.

### Analysis of SA-βgal positive cells in different types of colonies

**Figure 2:**
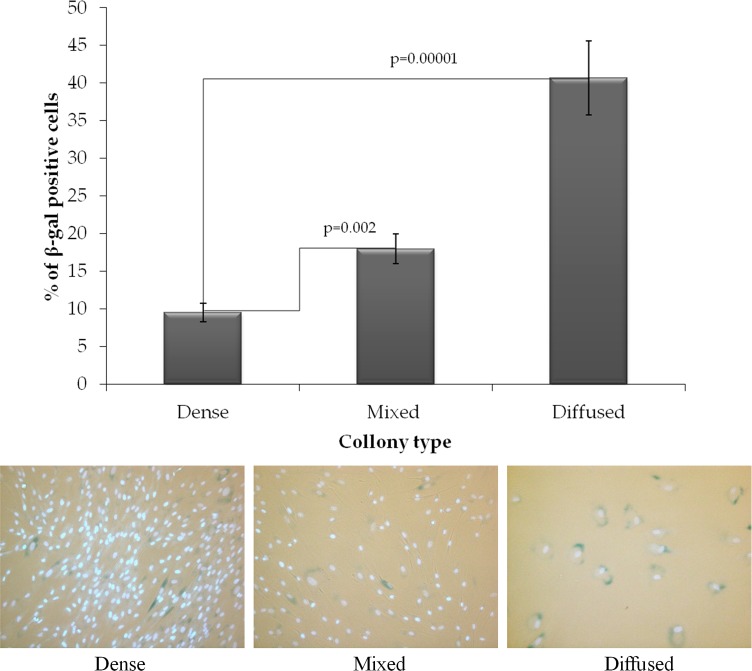
The fraction of SA-βgal positive cells in the colonies of various types Top panel, the bar plot of mean values of 10 donors studied ± SEM. Bottom panel, representative images of the aging cells (cytoplasm colored dark blue) in each type of colonies. Nuclei were counterstained with Hoechst 33342 (bright light blue).

Cells from different types of colonies had different morphological appearance, with those in diffuse colonies reminiscent of senescent cells. We therefore determined the fraction of SA-βgal+ cells in all three types of colonies of human skin fibroblasts. Indeed, the highest fraction of SA-βgal+ cells (40.6±4.9%) was found in the diffuse colony phenotype, whereas the dense colonies contained the lowest fraction of such cells (9.6±1.2%) (Figure 2). Both mixed and diffuse colonies contained significantly (p=0.002 and p=0.0001, respectively) higher fraction of SA-βgal+ cells. This observation is consistent with the proposed subdivision of human skin fibroblasts into three types [10]. Therefore, the fraction of diffuse colonies may be representative of the fraction of cells that are close to exhausting their proliferative capacity.

### Relationship of diffuse colonies to proliferation and degree of senescence

**Figure 3:**
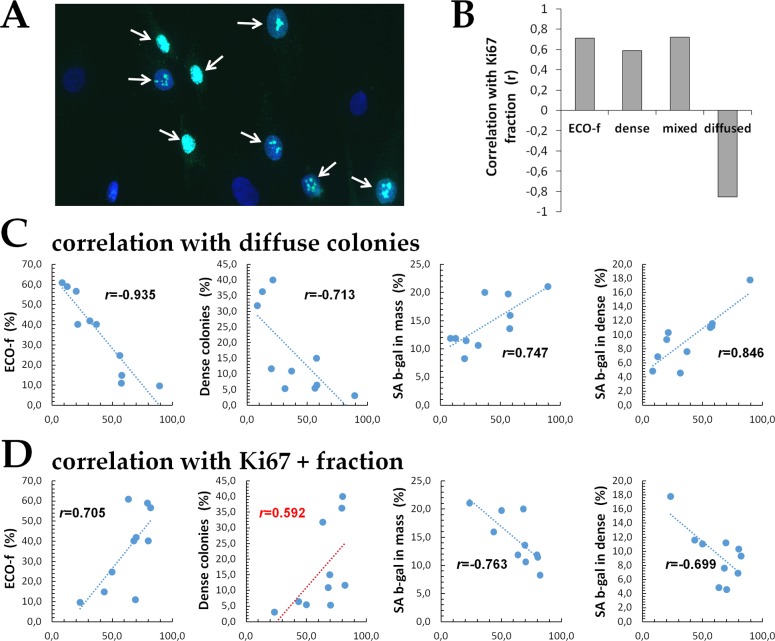
Correlation analysis (**A**) Representative microphotographs of fibroblasts immunofluorescently labeled for Ki67. (Ki67+ cells are marked with arrows), (**B**) The fraction of Ki67+ cells in mass culture vs. four indicated clonogenic endpoints; all correlations, except for a correlation with the dense colony fraction (p=0.07), were statistically significant at p<0.05, (**C**) The fraction of diffuse colonies (X-axis) vs. the other endpoints (Y-axis, as indicated). **(D**) The fraction of Ki67+ cells (X-axis) in mass culture and the other endpoints (Y-axis, as indicated). Note: Red color indicate a trend-line with the *r* value that is not statistically significant (p=0.07). The *r* values colored in black are statistically significant (*p*<0.05).

To examine the relationship of the fraction of diffuse colonies to proliferation in a fibroblast culture, we measured the fraction of proliferating cells in mass cultures using the Ki67 marker, which is in our opinion an excellent marker for quantification of the growth fraction of a given cell population (Figure 3A). The fractions of Ki67+ cells were quantified and are presented in Table 3. In parallel, to assess the level of cellular aging in cultures of human fibroblasts, we measured the fraction of SA-βgal+ cells in mass cultures (Table 3). The fractions of both of SA-βgal+ and Ki67+ cells failed to correlate (r=−0.17; p=0.32 and r=0.18; p=0.31, respectively) with the chronological age of the human donors. However, since the donors participated in this study may be grouped into a single age category, the range of the ages may not be great enough to detect a possible correlation of SA-βgal+ cells with chronological age. A correlation between SA-βgal+ and Ki67+ cells was revealed (r=−0.77; p=0.01), which was expected and simply reflects the fact that senescent cells lose their ability to proliferate. Subsequently, we examined the correlation between the fraction of Ki67+ cells and the four clonogenic endpoints (Figure 3B). We found the highest statistical degree of inverse correlation between the Ki67+ fraction and the fraction of diffuse colonies (r=−0.85, p=0.02) (Figure 3B). In contrast, for the rest of the clonogenic endpoints (ECO-f, the fraction of dense colonies and the fraction of mixed colonies), a strong positive correlation was observed (r=0.71, р=0.02; r=0.59, р=0.07; r=0.72, р=0.02, respectively). Further we examined correlations between the colony phenotypes and other endpoints related to fibroblasts proliferation or their degree of aging (Figure 3C). Statistically significant (p=0.0001) inverse correlation between the fraction of diffuse colonies and the clonogenic capacity was found, whereas the first endpoint demonstrated a significant positive correlation with the fraction of SA-βgal+ cells in both mass culture and dense colonies (р=0.013 and p=0.002, respectively). Dense colonies are in essence a monolayer of cells and the percentage of SA-βgal+ cells in them may be reflective of an overall aging status of a mass culture. This data suggests that the fraction of diffuse colonies may be informative for evaluating the degree of aging of fibroblast cultures. Lastly, we examined whether the fraction of Ki67+ cells in mass cultures correlated with the same four endpoints (Figure 3D). Mirror correlation patterns to those for diffuse colonies (Figure 3D) were observed, with one exception for the fraction of dense colonies which was not statistically significant (р=0.07).

**Table 3:** Analysis of SA-βgal+ and Ki67+ cells in mass cultures of human skin fibroblasts from 10 human donors

Patient ID	Patient age	SA-βgal+ cells in mass cultures, %	K67+ cells in mass cultures, %
1	38	11.9±1.6	79.5±8.1
2	33	8.3±1.1	82.1±6.4
3	33	19.7±2.1	50.0±3.7
4	54	13.7±1.7	69.6±5.4
5	48	11.9±1.5	63.6±7.7
6	34	21.1±2.4	23.4±1.9
7	43	20.0±1.9	68.2±9.3
8	46	16.0±1.8	43.4±3.9
9	43	11.5±1.3	80.1±5.7
10	41	10.7±0.9	70.2±6.8

Values are means ± SEM calculated from three measurements

## DISCUSSION

Our data may indicate that dense colonies that have a small number of β-galactosidase positive cells are the progeny of the fibroblast progenitor cells with high proliferative potential that can match MF I, while diffuse colonies with a significantly larger number of β-galactosidase positive cells, may be the descendants of less dividing MF III cellular pool at the 11 stage of differentiation sequence in the fibroblast equivalent stem cell system *in vivo*.

Gradual loss of cellular functions including the ability to proliferate with the increase in the number of cell divisions during cell population development was called “replicative aging” or “cellular senescence” [20]. It was previously shown that replicative aging indirectly leads to over-expression and accumulation of endogenous lysosomal β-galactosidase in cells with the progressing differentiation phenotype associated with aging [21]. Although a causative relationship between aging and SA-βgal is not clear [21] and evidence exists that SA-βgal is not a specific marker for senescence [22], it remains the most widely used marker to measure aging in human skin fibroblasts [23, 24]. The strong inverse correlation between the SA-βgal+ и Ki67+ human fibroblasts revealed in this study supports the use of SA-βgal staining for the evaluation of cellular aging. Indeed, by definition, the main criterion of cell aging is the loss of the cell's ability to divide [25, 26]. The use of both SA-βgal+ and Ki67+ for the evaluation of aging has been steadily growing in recent years [27]. Consequently, our data suggests that the MF III pool of skin cells that presumably reside in diffuse colonies have features of cellular senescence. Moreover, the fraction of cells that can form diffuse colony phenotype inversely correlated with the fraction of proliferating cells directly measured as the percentage of Ki67+ cells in a growing cell culture. Notably, among all the endpoints obtained by the clonogenic analyses, diffuse colonies showed the strongest correlation, making this measure a promising marker to evaluate a degree of cellular senescence in human skin fibroblasts.

Replicative aging leads to collagenase over-expression and loss of control of collagenase activity in human skin fibroblasts [28] with subsequent changes in the structure of intercellular matrix and the decreased ability to form intercellular contacts [29, 30]. In turn, these changes may be responsible for changes in the topology of the colonies and the transition to the diffuse type of cell location in colonies with high proportion of MF III cells. The features of cellular senescence expressed by skin cells might be attributed to CCN1 protein-mediated regulation associated with the replicative aging [31]. In addition, it was shown that, depending on the degree of replicative aging, the CCN1 activity level may play a significant role in the formation of fibrosis in wound healing and hence contribute to scarring and other skin blemishes [32]. Thus, the level of cell senescence of skin cell population is an important marker of direct clinical importance for the development of optimal individual skin care protocols. In view of the foregoing, it can be concluded that a simple analysis of the morphology of colonies during clonogenic test allows for evaluation of the degree of fibroblasts senescence in human skin samples. In particular, a higher degree of aging associated phenotype could be expected for cell cultures that form substantial number of diffuse phenotype colonies.

## METHODS

### Fibroblast isolation and culturing

All human donors provided written consent for this study. Ethics approval for the study was granted by the the Russian Healthcare Regulation Authority and the Ethics Committee and Academic Council of the Central Research Institute of Dental and Maxillofacial Surgery (#4/276). A skin biopsy was obtained with a disposable punch from behind the ear auricle under local infiltrative anesthesia with the 2% lidocaine solution. The isolated skin fragment (appx. 4 mm^3^) was immediately transferred to the labeled sterile container with shipment medium (DMEM/F12). Thereafter the biomaterial was transferred to a Petri dish under laboratory sterile conditions and washed three times with Hank's solution supplemented with antibiotic (gentamycin) in Gibco^®^ Versene solution (0.2 g EDTA(Na_4)_ per liter of Phosphate Buffered Saline (PBS), pH 7.4) manufactured by Thermo Fisher Scientific, USA. The tissue homogenate was prepared by sequential dispersing with a sterile scalpel, the addition of the disaggregating 0.1% collagenase solution, and the incubation of disaggregating solution at 37°C for 1-1.5 hours.

After the incubation, the tissue suspension was vigorously mixed by the pipette and centrifuged at 300 g for 10 minutes. The supernatant was discarded, and the pellet was resuspended again in the tissue culture medium (DMEM/F12, 1:1) containing 10% defined bovine fetal serum (FBS) (HyClone, USA) and 40 ug/ml of gentamicin (TCM). The resulting suspension was transferred into a culture vial and incubated under the 5% CO_2_ atmosphere at 37°C in CO_2_-incubator. The culture medium was changed every 3-4 days.

At 70% of confluency, the cells were washed with Gibco^®^ Versene solution and detached from the surface of a culture flask by use of Gibco^®^ Versene solution and 0.25% trypsin at 37°C for 3-5 minutes. The cell suspension was diluted in a culture medium and explanted into a larger cultural flask for subsequent cultivation.

### Clonal fibroblast analysis

At second cell passage, the cell monolayer was washed three times with Gibco^®^ Versene solution and trypsinized at 37°C, 5% CO_2_ for 10 min. The homogenate was centrifuged at 300 g for 10 minutes. The supernatant fluid was discarded, cells were resuspended in Hank's solution, and counted in Gorjaev's chamber.

The cells were diluted by TCM to obtain final concentration 100 cells/ml. The cell suspension was split into three 100 mm Petri dishes to obtain a clonal inoculum density of 1.5 cells/cm^2^.

The Petri dishes were incubated in a CO_2_-incubator at the saturated humidity conditions at 37°C in the 5% CO_2_ atmosphere for 14 days. Thereafter, the culture dishes with the pre-formed colonies were washed three times with PBS (рH 7.4 and fixed with 70% alcohol for 15 minutes at room temperature. Then alcohol residuals were removed by triple washing with distilled water, and the colonies were stained with a KaryoMAX® Giemsa Stain Stock Solution produced by Gibco, USA, for 20 minutes at 37°C. The dishes with the stained colonies were thoroughly washed from excessive stain and dried at room temperature for 5-7 hours.

Thereafter, the analysis of the colonies was carried out. All of the colonies were ranked in three groups: dense, diffuse and mixed colonies. The colony forming effectiveness of the fibroblasts (ECO-f) was determined according to Fridenshtein's equation for stromal progenitor cells: ECO-f = a ratio between the number of pre-formed colonies and the number of explanted cells multiplied by 100% [7, 33]. The only counted colonies include more than 20 cells of the total number of explanted cells. The clones of fibroblasts which include fewer cells were not considered as colonies and, accordingly, were not included in the count.

### Analysis of β-galactosidase positive cells

To quantify the proportion of β-galactosidase positive cells the commercial kit “Cellular Senescence Assay» (EMD Millipore, USA, Catalog Number: KAA002) was used. The cells were stained according to supplemented manufacturer protocol with the following modification: at the final РВS washing step, the cell nuclei were stained with 1 μg/ml Hoechst 33342 (Molecular Probes, USA). Such modification significantly improves the quality of counting of β-galactosidase negative cells (Figure 2). The stained cells were visualized using Excitation/Emission Interference Filters (СKX-U: 340-380nm/435-485 nm) on the inverted fluorescent microscope Оlympus СКХ 41 SF (Оlympus, Japan) equipped with Infinity 3-1 (Lumenera Copr., Canada) CCD camera and 20X objective.

### Immunocytochemical analysis of Ki67 positive cells

For immunocytochemical analysis cells were seeded at the density of 5 × 10^3^ cells/cm^2^ in 500 μL of culture medium onto coverslips placed inside 35 mm Petri dishes (Corning, USA). To improve adhesion of cells additional volume of culture medium (1.5 mL) was added into Petri dishes 15 minutes after seeding. Cells seeded on coverslips were incubated at 37°C and 5% CO_2_ for at 48 h prior to fixation.

Cells were fixed on coverslips in 4% paraformaldehyde in PBS (pH 7.4) for 15 min at room temperature followed by two rinses in PBS and permeabilization for 40 min with 0.3% Triton-X100 (in PBS, pH 7.4) supplemented with 2% bovine serum albumin (BSA) to block non-specific antibody binding. Cells were then incubated for 1 hour at room temperature with primary mouse monoclonal antibody against Ki67 protein (dilution 1:400, clone Ki-S5, Merck-Millipore, USA) which were diluted in PBS with 1% BSA. Following several rinses with PBS, cells were incubated for 1 hour at room temperature with secondary antibodies IgG (H+L) goat anti-mouse (Alexa Fluor 488 conjugated, dilution 1:600; Merck-Millipore, USA) with 1% BSA. Coverslips were then rinsed several times with PBS and mounted on microscope slides with ProLong Gold medium (Life Technologies, USA) with DAPI for DNA counter-staining. Cells were viewed and imaged using Nikon Eclipse Ni-U microscope (Nikon, Japan) equipped with a high definition camera ProgRes MFcool (Jenoptik AG, Germany). Filter sets used were UV-2E/C (340–380 nm excitation and 435–485 nm emission) and B-2E/C (465–495 nm excitation and 515–555 nm emission). At least 200 cells per data point were counted.

### Statistical analysis

Statistical and mathematical analyses of the data were conducted using the Statistica 8.0 software (StatSoft). Data points in Figures are mean values obtained from three independent experiments; error bars are standard errors. Statistical significance was tested using the Student t-test at p < 0.05.
